# The incremental economic burden of heart failure: A population-based investigation from South Korea

**DOI:** 10.1371/journal.pone.0208731

**Published:** 2018-12-21

**Authors:** Hyeonseok Cho, Sung-Hee Oh, Hankil Lee, Hyun-Jai Cho, Hye-Young Kang

**Affiliations:** 1 College of Pharmacy, Yonsei Institute of Pharmaceutical Sciences, Yonsei University, Incheon, South Korea; 2 Division of Cardiology, Department of Internal Medicine, Seoul National University Hospital, Seoul, South Korea; Massachusetts General Hospital, UNITED STATES

## Abstract

**Background:**

The prevalence of heart failure (HF) and its economic burden are increasing with age of the South Korean population. This study aimed to assess the economic impact of HF from the societal perspective.

**Methods:**

A prevalence-based, incremental cost-of-patient study was performed to estimate the cost ratio between patients with HF and those without HF based on the claims database of the national health insurance in South Korea. We defined adult HF patients as those aged ≥19 years who had at least one insurance claim record with a primary or secondary diagnosis of HF. Age- and gender-matched controls were defined using a 1:4 greedy matching method. Costs were estimated by including medical costs for insurance-covered and non-covered services, transportation costs, caregiver’s cost, and time costs of patients. The ratio of costs between patients with HF and those without HF was adjusted for age, gender, and type of universal health security program in the multivariate regression model.

**Results:**

The average annual per-capita cost was estimated to be $6,601 for patients with HF (n = 14,252), which is about 3.38 (95% confidence interval [CI]: 3.31–3.46) times higher than that for patients without HF (n = 1,116,882) and 1.64 (95% CI: 1.59–1.70) times higher than that for the age- and gender-matched patients without HF (n = 57,008). In the multivariate regression model, the annual per-capita total costs were 1.98-fold (95% CI: 1.94–2.02) statistically higher for patients with HF than for patients without HF after adjustment for age, gender, and type of universal health security program.

**Conclusions:**

This study demonstrates a significant incremental burden of HF. Given that the prevalence of HF is expected to increase with an increase in the aging population, the national economic burden is expected to be substantial in the future. Thus, greater emphasis on the prevention and treatment of HF is warranted.

## Introduction

Defined as “a complex clinical syndrome that results from any structural or functional impairment of the ventricular filling or ejection of blood” [1], heart failure (HF) is a progressive disease with repeated recurrences resulting in frequent hospitalization and high costs [2]. The epidemiologic burden of HF is substantial, and it has been estimated that about 26 million people are affected worldwide [3]. Since the incidence of HF is associated with aging [4], the global burden of HF is expected to grow, particularly in regions facing accelerated increase in the elderly population. In South Korea, a country that is experiencing a rapidly aging population, the prevalence of HF has increased progressively in the recent years, approximately two times from 0.75% in 2002 to 1.53% in 2013 [5]. The other important risk factor for HF is ischemic heart disease, which is also associated with aging and expected to increase due to adoption of western lifestyle [5]. In addition, HF is one of the leading causes of death in South Korea [6].

A number of studies have investigated the economic burden of HF. In Europe and USA, 1–2% of the annual healthcare budget was attributable to HF [7]. In Sweden, the annual national cost of HF was estimated to be SEK 2.0–2.6 billion, representing about 2% of the total public healthcare budget [8]. The direct medical costs incurred by HF patients accounted for 1.9% of the total National Health Service budget in England [9]. The annual treatment cost for a patient with HF in China was RMB 28,974 in 2014 [10]. From a societal perspective, Lee et al. estimated that the annual economic burden of treating HF ranged from $1,414 to $1,561 in South Korea in 2014 [11].

Most of these earlier studies calculated the economic burden as the expenditure resulting from the treatment of HF and presented it as either per-capita or the total nationwide cost of treatments [7–11]. While such information is useful to estimate the absolute amount of spending on the treatment of HF patients, it is limited in that the excess costs attributable to the presence of HF cannot be estimated. An incremental cost-of-patient approach overcomes this limitation by comparing the relative costs between patients with HF and those without HF represented by the ratio of costs.

This study aimed to examine the economic impact of HF on individual patients and society by assessing the cost ratio between patients with HF and those without HF based on the claims database of the national health insurance in South Korea.

## Methods

### Study design and data source

The economic impact of HF was assessed using a cost ratio between patients with HF and those without. To calculate costs during the study period, an all-cause approach was selected. While the disease-specific approach focuses on measuring the costs incurred by certain disease categories [12], all potential costs, whether directly or indirectly related to the major disease (i.e., HF), can be captured under the all-cause approach by avoiding restriction to certain disease categories [13,14].

To identify patients with HF and examine their health care utilization, we used the 2014 Health Insurance Review and Assessment Service–National Patients Sample (HIRA-NPS) data, which is nationally representative and open to the public for research purposes. The HIRA–NPS data are cross-sectional, available for each year since 2010, and composed of health insurance claim records during the year. There are about 1,400,000 individuals in the database drawn by 3% random stratified sampling from the entire population who have a claim record during the year. South Korea has a government-run mandatory national health security system composed of 97% enrolled in the National Health Insurance (NHI) and 3% in Medical Aid (MA) programs. The HIRA-NPS data cover both the NHI and MA beneficiaries and provide claim records for all types of insurance-covered services including inpatient and outpatient care, emergency room visits, diagnostic procedures, and prescription medication used by beneficiaries including socioeconomic variables such as age, gender and type of universal health security program. This study was approved by the Yonsei University Institutional Review Board (IRB No. 1040917-201601-SB-107-02E), and the requirement for informed consent was waived since the analysis was based on secondary data.

### Study subjects

Cases were defined as adult HF patients aged 19 years and above who had at least one insurance claim record and a primary or secondary diagnosis of HF according to the 2014 HIRA-NPS data. Based on the literature review [15–19], we identified the diagnostic codes (International Classification of Disease 10^th^ Revision (ICD-10 codes)) for HF: I11.0 (hypertensive heart disease with [congestive] heart failure), I13.0 (hypertensive heart and renal disease with [congestive] heart failure), I13.2 (hypertensive heart and renal disease with both [congestive] heart failure and renal failure), or I50.x (heart failure).

Controls were defined as non-HF patients aged 19 years and above who did not have any insurance claim record and a diagnosis of HF according to the 2014 HIRA-NPS data. Age- (within 5 years) and gender-matched controls were also defined using a 1:4 greedy matching method [20,21]. For each study subject included in the case, control, or matched control groups, all claim records from each individual processed in 2014 were retrieved to calculate the annual total costs per person.

### Estimating the incremental costs of HF

The economic burden of HF was estimated using an all-cause approach by calculating the ratio of costs for all conditions, regardless of the type of disease, between patients with HF and those without. Using the societal perspective, costs were estimated by including the medical costs for insurance-covered and non-covered services, transportation costs, caregiver’s cost, and time costs of patients.

For each study subject, the total annual medical costs for the insurance-covered services were calculated by adding all medical costs reported in claim records in 2014. Using the ratio of non-insurance-covered to insurance-covered costs (i.e., 0.19:0.81) among patients with heart disease, the total annual medical costs for non-insurance-covered services were estimated for individual patients with HF [22]. For patients without HF, the ratio of 0.21:0.79 observed among patients with all types of disease was applied [22]. The annual per-capita transportation costs were estimated by multiplying the total number of outpatient visits and hospital admissions per year by the average two-way transportation costs to visit the healthcare institutions, which were obtained from the 2006 Korean National Health and Nutrition Examination Survey [23] (Table A in S1 File). Caregiver costs were computed as the product of the total annual inpatient days and the average daily rate for a helper [24] (Table B in S1 File). These costs were converted to the 2014 values using the consumer price index for public transportation and care services [25]. Patients’ time costs due to morbidity were measured as the productivity loss cost during hospitalization and outpatient visits based on the human capital approach (Eq 1) [26,27]. While the other input data were estimated from HIRA-NPS-2014, the employment rate and average daily income by age and gender were obtained from the Korean Statistical Information Service [28,29], and the average hours per outpatient visit were referenced from the Korean National Health and Nutrition Examination Survey [23] (Table C in S1 File). Based on the assumption that those aged 65 and above are no longer in the labor market, we applied the productivity loss costs only for those patients younger than 65 years old.

$$\begin{array}{l} \mathrm{Productivity}\;\mathrm{loss}\;\mathrm{costs}\;\mathrm{due}\;\mathrm{to}\;\mathrm{morbidity}=\sum_{i}\sum_{j}\left\{\left(I_{ij}\times D_{ij}\times P_{ij})+(O_{ij}\times V\times H_{ij}\times P_{ij}\right)\right\}\\ \mathrm{Here},\\ i=\mathrm{age}\\ j=\mathrm{gender}\\ I_{ij}=\mathrm{average}\;\mathrm{annual}\;\mathrm{inpatient}\;\mathrm{days}\;\mathrm{per}\;\mathrm{patient}\;\mathrm{with}\;\mathrm{HF}\;(\mathrm{or}\;\mathrm{without}\;\mathrm{HF})\;\mathrm{by}\;\mathrm{age}\;\mathrm{and}\;\mathrm{gender}\\ D_{ij}=\mathrm{average}\;\mathrm{daily}\;\mathrm{income}\;\mathrm{by}\;\mathrm{age}\;\mathrm{and}\;\mathrm{gender}\\ P_{ij}=\mathrm{employment}\;\mathrm{rate}\;\mathrm{by}\;\mathrm{age}\;\mathrm{and}\;\mathrm{gender}\\ O_{ij}=\mathrm{average}\;\mathrm{annual}\;\mathrm{number}\;\mathrm{of}\;\mathrm{outpatient}\;\mathrm{visits}\;\mathrm{per}\;\mathrm{patient}\;\mathrm{with}\;\mathrm{HF}\;(\mathrm{or}\;\mathrm{without}\;\mathrm{HF})\;\mathrm{by}\;\mathrm{age}\;\mathrm{and}\;\mathrm{gender}\\ V=\mathrm{average}\;\mathrm{hours}\;\mathrm{per}\;\mathrm{outpatient}\;\mathrm{visit}\\ H_{ij}=\mathrm{average}\;\mathrm{hourly}\;\mathrm{wage}\;\mathrm{by}\;\mathrm{age}\;\mathrm{and}\;\mathrm{gender} \end{array}$$ Eq 1

The following characteristics and use of healthcare resources among the patients with HF and those without, which may help to understand the potential difference in total costs, were examined: the average age, gender distribution, distribution of type of universal health security program, the annual per-capita number of outpatient visits, hospital admissions, and inpatient days. In addition, the Charlson Comorbidity Index (CCI) score, the annual per-capita tertiary-care hospital visits, and distribution of patients experiencing hospital admission were estimated as a proxy measure of severity. CCI score is widely used to evaluate each patient’s baseline condition with a higher score indicating a worse health status [30]. To evaluate the severity of the patients’ condition other than HF, we calculated the CCI score excluding HF, the annual per-capita tertiary-care hospital visits, and distribution of patients experiencing hospital admission with primary causes other than HF.

### Statistical analysis

Testing for statistical differences in patient characteristics and healthcare resource utilization between subgroups was performed using the Chi-square test for the categorical variables, and the *t*-test for the continuous variables. To elucidate the impact of HF on the total costs while adjusting factors that affect them, multiple regression analyses were conducted. Based on the literature review [31–33], generalized linear model (GLM) with log link and gamma distribution was selected to account for the right-skewed distribution of the cost data. The dependent variable was the total cost for patients with HF and those without HF, and the independent variable was the presence of HF (cases vs. non-matched controls). We included the following confounding variables for the regression model to adjust for the socioeconomic status: the age group (<50, 50–59, 60–69, 70–79, >80), gender, and type of universal health security program (NHI, MA). The model fittings were tested based on the Akaike information criterion (AIC), while the correlations among the explanatory variables were measured using the variance inflation factor (VIF). A p-value < 0.05 was considered statistically significant. All statistical analyses were performed using the SAS software, version 9.4 (SAS Institute, Inc., Cary, NC, USA).

## Results

### The estimated prevalence and characteristics of HF patients

Among 1,152,073 adult patients in the HIRA-NPS-2014 database, 14,252 patients were identified as having HF, so that the estimated prevalence of HF was 1,237 per 100,000 adult population. A total of 544,999 claim records for any disease or medical conditions were identified from 14,252 adult HF patients based on the 2014 HIRA-NPS data. Among 544,999 claims, 74,865 claims (13.74%) were considered directly associated with HF because they had HF (ICD-10 code: I11.0, I13.0, I13.2, or I50.x) as a primary or secondary diagnosis. For the control group without any claim records containing HF diagnostic codes, 1,116,882 individuals were identified with 18,419,350 claim records with any disease or medical conditions from the 2014 HIRA-NPS data. For the 1:4 age- and gender-matched control group, 57,008 individuals with 1,598,152 claim records with any disease or medical conditions were identified.

HF patients showed a smaller proportion of men (42.31% vs. 47.86%, p-value < 0.0001) and greater mean age (68.43 vs. 45.90 years old, p-value < 0.0001) as compared to those in the control group (Table 1). The proportion of the elderly, aged 65 years and above, among the HF patients (64.62%) was about 4.4 times greater than that among the control group (14.57%) (p-value < 0.0001), and this difference was statistically significant. The proportion of patients with HF enrolled in the MA program (10.27%) was statistically significant, and about 3.8 times higher than that of the patients without HF (2.69%) (p-value < 0.0001).

**Table 1:** Demographic and healthcare utilization characteristics of the study subjects.

Characteristics	Cases (HF patients)	Controls (non-HF patients)	Matched controls^e^	p-value (cases vs. controls)	p-value (cases vs. matched controls)
No. of adult patients (≥19 years old)	14,252	1,116,882	57,008	-	-
Male, %	42.31	47.86	42.31	<0.0001	1.0000
Age, mean (SD)	68.43 (13.21)	45.90 (16.41)	68.43 (13.21)	<0.0001	0.9989
Elderly (≥65 years old), %	64.62	14.57	65.12	<0.0001	0.2697
Patients enrolled in the Medical Aid program, %	10.27	2.69	6.24	<0.0001	<0.0001
Charlson Comorbidity Index score, mean (SD)	4.07 (2.48)	0.96 (1.50)	1.87 (2.05)	<0.0001	<0.0001
Charlson Comorbidity Index score excluding HF^a^, mean (SD)	3.11 (2.50)	0.96 (1.50)	1.87 (2.05)	<0.0001	<0.0001
No. of outpatient visits per patient per year, mean (SD)^b^	37.10 (34.87)	16.20 (19.97)	27.33 (28.81)	<0.0001	<0.0001
No. of hospital admissions per patient per year, mean (SD)^b^	1.14 (2.52)	0.29 (1.31)	0.70 (2.25)	<0.0001	<0.0001
No. of inpatient days per patient, mean (SD)^b^	15.11 (49.03)	3.64 (26.87)	12.24 (54.29)	<0.0001	<0.0001
Patients experiencing hospital admission, %	39.11	13.75	22.20	<0.0001	<0.0001
Patients experiencing hospital admission due to primary causes other than HF ^c^, %	34.23	13.75	22.20	<0.0001	<0.0001
No. of tertiary-care hospital visits^d^ per patient, mean (SD)	3.01 (8.28)	0.84 (3.75)	1.38 (4.90)	<0.0001	<0.0001
No. of tertiary-care hospital visits^d^ per patient due to primary causes other than HF ^c^, mean (SD)	2.40 (7.50)	0.84 (3.75)	1.38 (4.90)	<0.0001	<0.0001

All characteristics listed correspond to the results for adult patients aged 19 years or above unless otherwise specified.

^a^ Excluding scores derived from diagnosis of HF (ICD-10 codes of I11.0, I13.0, I13.2, or I50.x).

^b^ Healthcare utilization due to all causes.

^c^ Without a primary or a secondary diagnosis of HF (ICD-10 codes: I11.0, I13.0, I13.2, or I50.x).

^d^ Including both inpatient and outpatient visits.

^e^ 1:4 matching by age (±5 years) and gender.

HF, heart failure; SD, standard deviation

The average CCI score was higher among the HF patients (4.07) than that among the control (0.96) and age- and gender-matched control patients (1.87). For individual study subjects, a total CCI score was computed by adding the CCI scores for the diagnostic codes included in the claim records for all types of health care services during the study year of 2014. To examine the baseline health status excluding the impact of HF, we also compared the CCI scores, excluding scores derived from the HF diagnosis. As a result, the average CCI score for other health conditions among the HF patients decreased from 4.07 to 3.11 but remained higher compared to those of the control (0.96) and age- and gender-matched control patients (1.87).

As compared to the control patients, HF patients showed increased utilization of both outpatient and inpatient services by 2.29 folds (37.1 vs. 16.2 outpatient visits) and 3.93 folds (1.14 vs. 0.29 admissions), respectively. This gap decreased by 1.36 folds (37.1 vs. 27.33 outpatient visits) and 1.63 folds (1.14 vs. 0.70 admissions) when compared to that among the age- and gender-matched control patients, respectively. For the per-capita annual inpatient days, the gap was substantially lesser than those of the age- and gender-matched control patients (1.23 folds: 15.11 vs. 12.24 days) and those of the control patients (4.15 folds: 15.11 vs. 3.64 days). The proportion of patients experiencing hospital admission was statistically higher among patients with HF than those with the age- and gender-matched controls (39.11% vs. 22.20%, p-value < 0.0001). Tertiary-care hospitals were used 2.18 folds (3.01 vs. 1.38) more frequently by the HF patients than by the age- and gender-matched control patients for any disease or medical conditions, including inpatient and outpatient services. The severity of the patients’ condition other than HF, measured using the annual per-capita tertiary-care hospital visits and distribution of patients experiencing hospital admission due to primary causes other than HF, was also statistically higher among patients with HF than that among the age- and gender-matched controls (p-value < 0.0001).

### Incremental costs of HF patients

The average annual per-capita insurance-covered medical costs for HF patients were $4,194 (1 US dollar approximately equals to 1,100 Korean won) (Table 2). Within this expense, $887 (21.1% of $4,194) appeared to be directly associated with HF, as the primary or secondary diagnosis on the claim records was HF. For the control and age- and gender-matched control groups, the average annual per-capita insurance-covered medical costs were $1,063 and $2,367, respectively, leading to a cost ratio of 3.94 (case vs. control) and 1.77 (case vs. age- and gender-matched control) (Table 2). The difference in the cost between the HF patients and the age- and gender-matched control patients was $1,827 (= $4,194-$2,367), of which $887 was directly attributed to HF, as mentioned above.

**Table 2:** Average annual cost per patient with heart failure and without heart failure in 2014.

	Per-capita cost, USD				
	Cases (HF patients)	Controls (non-HF patients)	Matched controls^b^	Ratio of costs (cases vs. controls)	Ratio of costs (cases vs. matched controls^b^)
Direct costs	6,215	1,653	3,823	3.76	1.63
Medical costs	4,990 (75.6%)	1,287 (65.9%)	2,864 (71.3%)	3.88	1.74
Insurance-covered	4,194	1,063	2,367	3.94	1.77
Inpatient services (including prescription drug costs)	2,193	422	1,194	5.20	1.84
Outpatient services (including prescription drug costs)	2,001	641	1,172	3.12	1.71
Prescription drug costs^a^	817	207	462	3.95	1.77
Non-covered	797	223	497	3.57	1.60
Non-medical costs	1,233 (18.3%)	368 (17.9%)	966 (23.7%)	3.35	1.28
Transportation costs	391	166	285	2.36	1.37
Caregiver’s costs	833	201	675	4.16	1.24
Indirect costs	386 (5.8%)	299 (15.3%)	191 (4.8%)	1.29	2.02
Productivity loss due to morbidity	386	299	191	1.29	2.02
Total costs (direct + indirect costs)	6,601 (100.0%)	1,952 (100.0%)	4,014 (100.0%)	3.38	1.64

1 USD = 1,100 Korean won; USD, U.S. dollar

^a^ Prescription drug costs were calculated as the sum of inpatient and outpatient prescription drug costs.

^b^ 1:4 matching by age (±5 years) and gender.

To speculate the potential causes for the remaining difference of $940 (= $1,827-$887), we examined the distribution of the 10 most frequent primary diagnoses of claim records among the age- and gender-matched control group, which was then compared with the distribution of the 10 most frequent primary diagnoses of claim records unrelated to HF (i.e., claim records without having HF as a primary or secondary diagnosis) among the case group (Table 3). The distribution of the diagnoses between the two groups was very similar: nine of 10 diagnoses were identical between the two groups and the proportion of each diagnosis was also very similar between the two groups. However, the proportion of healthcare use with a primary diagnosis of chronic kidney disease was about four times statistically higher among HF patients (4.68%) than that among the age- and gender-matched control patients (1.02%), which was not included in the list of the top 10 diseases of the matched control group. Because healthcare services for chronic kidney disease, such as dialysis, are expensive, it is considered that the part of the incremental cost of HF patients is due to the higher prevalence rate of chronic renal failure among the HF patients.

**Table 3:** Comparison of primary diagnosis of healthcare utilization unrelated to HF between HF and non-HF patients.

Rank	Cases (HF patients)		Matched controls ^a^ (non-HF patients)	
	Description (ICD-10 codes)	Proportion (%)	Description (ICD-10 codes)	Proportion (%)
1	Gonarthrosis [arthrosis of knee] (M17)	5.19	Essential (primary) hypertension (I10)	10.29
2	Dorsalgia (M54)	5.10	Dorsalgia (M54)	5.44
3	Essential (primary) hypertension (I10)	4.86	Gonarthrosis [arthrosis of knee] (M17)	5.12
4	Chronic kidney disease (N18)	4.68	Type 2 diabetes mellitus (E11)	3.42
5	Other spondylopathies (M48)	3.22	Other spondylopathies (M48)	2.88
6	Type 2 diabetes mellitus (E11)	2.73	Acute bronchitis (J20)	2.61
7	Acute bronchitis (J20)	2.65	Gingivitis and periodontal diseases (K05)	2.53
8	Other intervertebral disc disorders (M51)	2.16	Other intervertebral disc disorders (M51)	1.91
9	Gingivitis and periodontal diseases (K05)	2.01	Spondylosis (M47)	1.74
10	Spondylosis (M47)	1.68	Shoulder lesions (M75)	1.64

Listed above are primary diagnoses of healthcare utilization other than heart failure.

^a^ 1:4 matching by age (±5 years) and gender.

HF, heart failure; ICD-10, International Classification of Disease 10th Revision

To achieve a better understanding of the incremental costs of HF for subgroups according to patient age and the type of health care services provided, we compared the inpatient and outpatient insurance-covered medical costs across different age groups: <50, 50–59, 60–69, 70–79, and ≥80 years old (Figure A in S1 File). For inpatient services, both the HF and non-HF patient groups showed increase in costs with age. On the other hand, the medical costs spent for outpatient services increased up to the age of 70–79 years and began decreasing after 79. This trend was the same for both the HF and non-HF patients. Overall, the incremental costs of HF patients as compared to those of non-HF patients decreased with age, regardless of the type of health care services. However, the slope of the decrease was sharper for the outpatient services than that for the inpatient services.

The total economic burden of HF was computed from the societal perspective, adding the medical costs for the insurance-covered and non-covered services, transportation costs incurred by visiting the healthcare institutions, and the patients’ and caregivers’ time costs (Table 2). On an average, the annual economic burden of an adult HF patient was estimated to be $6,601 in 2014, while that of a control patient was $1,952, resulting in an incremental cost of $4,649 and a cost ratio of 3.38 (95% confidence interval [CI]: 3.31–3.46) in an HF patient. For the age- and gender-matched control patients, the annual per-capita burden was $4,014, resulting in an incremental cost of $2,587 and a cost ratio of 1.64 (95% CI: 1.59–1.70) in an HF patient.

In the multivariate regression analysis using the GLM, patients with HF exhibited statistically significantly higher total than patients without HF, after adjustment for age, gender, and type of universal health security program (Cost ratio: 1.98, 95% CI: 1.94–2.02) (Table 4).

**Table 4:** Regression analysis results showing comparison of annual per-capita total costs between patients with HF and those without HF using a generalized linear model.

	Ratio of costs (95% CI)
Heart failure	
No	ref
Yes	1.98 (1.94–2.02)
Age group	
<50	ref
50–59	2.00 (1.99–2.01)
60–69	2.63 (2.61–2.65)
70–79	3.66 (3.63–3.70)
≥80	5.25 (5.18–5.32)
Sex	
Male	ref
Female	0.95 (0.94–0.95)
Type of universal health security program	
National Health Insurance	ref
Medical Aid	4.59 (4.53–4.65)

HF, heart failure; CI, confidence interval; ref, reference category

The total national economic burden of HF in 2014 was calculated by multiplying the incremental cost of an HF patient ($2,587 or $4,649) by the total estimated number of HF patients in 2014. By applying sampling weight (33.33) to the samples, the total estimated number of HF patients in 2014 was 475,019. The resulting costs ranged from 1.23 to 2.21 billion dollars depending on whether the per-capita incremental cost of HF patients was calculated as compared to those for the non-HF patients or age- and gender-matched non-HF patients.

## Discussion

Our results have provided evidence on the impact of HF on the total costs based on the all-cause, incremental cost-of-patient approach. We confirmed statistically significant increase in utilization of healthcare resources by HF patients than that by patients without HF. Depending on whether the costs were compared between HF patients and non-HF patients or between HF and age- and gender-matched non-HF patients, patients with HF had 3.38 or 1.64 times higher total costs, respectively (Table 2). After adjustment for age, gender, and type of universal health security program, the total costs of patients with HF were estimated to show a statistically significant increase of 1.98-folds compared to that of patients without HF (Table 4).

There are several studies which have determined that patients with HF showed higher costs than those without HF. For example, a study conducted in the US reported a 4.33 times higher mean expenditure ($23,854) for patients with HF than that for patients without HF ($5,511), after adjustment for demographics and comorbidities [34]. Liao et al. (2007) also reported greater annual average costs for HF patients ($10,832) as compared to those of non-HF patients ($6,162), resulting in a cost ratio of 1.76 [35]. Chen et al. (2014) reported that patients with HF are associated with 1.255 times higher total healthcare costs than those in patients without HF (p-value < 0.001) based on a nationally representative sample of Medicare beneficiaries [36]. Comparisons of the results of our study with those of the previous studies should be interpreted with caution, since the definitions of HF and cost components considered in the analysis vary across studies. Nevertheless, earlier evidence from other countries has consistently shown significantly higher total costs in patients with HF than those in patients without HF.

On comparison of patient characteristics, the proportion of MA beneficiaries among patients with HF was 1.6 times statistically higher than that among the age- and gender-matched patients without HF. Hawkins et al. (2012) found that lower socioeconomic status was associated with an increased incidence of HF, which may explain the high proportion of MA beneficiaries among the patients with HF in our study [37]. The CCI score, excluding the score derived from the diagnosis of HF, was still higher among patients with HF, indicating that the baseline health status of patients with HF, irrespective of HF, was poor as compared to that of age- and gender-matched patients without HF. In addition, we found out that the proportion of chronic kidney disease was about 4 times statistically higher among HF patients than that among the age- and gender-matched control patients (Table 3). Ahmed et al. (2008) have reported the association between HF and chronic kidney disease that can be linked to the many shared risk factors [38]. Such differences in baseline health status contributed to the additional resource use by patients with HF, which may be attributed to higher total healthcare costs.

In the present study, HF patients had a higher mean age (Table 1) and showed higher medical costs for all age groups than did non-HF patients (Figure A in S1 File), which corresponds with the findings of previous research [2,5,8]. Notably, the incremental costs between the patients with HF and those without HF are decreasing with age. We can speculate that the impact of HF seems to be weakened by other comorbidities in the elderly patients because the risk for chronic disease increases with age.

There was a trend that the unadjusted average annual cost per patient was higher in patients with HF than that in patients without HF, though the estimated cost ratio were slightly different (Table 2). The high cost ratios were observed among the inpatient services and caregiver’s cost because of frequent hospitalization of patients with HF. On the other hand, the cost ratio for productivity loss due to morbidity showed relatively low value because the distribution of elderly patients, who are no longer in the labor market, was higher in these cases. These trends disappeared after age- and gender-matching.

There were some several limitations to this study. First, due to the lack of patient-level mortality data, the productivity loss costs due to premature death were not taken into account. Second, the employment rate was assumed to be the same for both patients with and without HF. However, the proportion of MA beneficiaries was higher among patients with HF, which might have contributed to the overestimated cost ratio. To estimate the true burden of HF, an integrated database providing sufficient data including etiology, mortality, and socioeconomic status is needed for future research.

## Conclusions

This study shows that the cost for patients with HF is significantly higher than that for patients without HF. Our findings estimated an incremental burden of 1.98 times the total healthcare cost for patients with HF. Given that the prevalence of HF is expected to increase with the rise in the aging population, the national economic burden of HF will be substantial in the future. Thus, a greater emphasis on the prevention and treatment of HF is warranted.

## Supporting information

S1 File (Table A) Input data for transportation costs per visit. (Table B) Input data for caregiver cost per day. (Table C) Input data for average daily income, hourly wage, and employment rate by age and gender. (Figure A) Incremental insurance-covered costs by age and the type of health care services.
(DOCX)
