# Supplementary material for: The incremental economic burden of heart failure: A population-based investigation from South Korea
Source: PLoS One. 2018 Dec 21;13(12):e0208731. doi: 10.1371/journal.pone.0208731 (PMC6303080; doi:10.1371/journal.pone.0208731)
Supplement: S1 File — (Table A) Input data for transportation costs per visit. (Table B) Input data for caregiver cost per day. (Table C) Input data for average daily income, hourly wage, and employment rate by age and gender. (Figure A) Incremental insurance-covered costs by age and the type of health care services. (DOCX) [file pone.0208731.s001.docx]

Table A. Input data for transportation costs per visit.

| Year | 2005 | 2014 |
| --- | --- | --- |
| Two-way transportation costs for outpatient visit (USD) | 7.6 | 9.8 |
| Two-way transportation costs for inpatient visit (USD) | 19.3 | 24.9 |
| Consumer price index for transportation | 83.823 | 108.10 |

1 USD = 1,100 Korean won; USD, U.S. dollar

Table B. Input data for caregiver cost per day.

| Year | 2008 | 2014 |
| --- | --- | --- |
| Daily rate for a helper (USD) | 47.9 | 55.1 |
| Consumer price index for care services | 97.43 | 112.24 |

1 USD = 1,100 Korean won; USD, U.S. dollar

Table C. Input data for average daily income, hourly wage, and employment rate by age and gender.

| Male | | | |
| --- | --- | --- | --- |
| Age (years) | Average daily income  (USD) | Average hourly wage  (USD) | Employment rate (%) |
| 19 | 67.8 | 7.8 | 6.8 |
| 20-24 | 77.2 | 8.7 | 39.4 |
| 25-29 | 98.6 | 11.3 | 69.4 |
| 30-34 | 119.7 | 13.9 | 89.8 |
| 35-39 | 141.2 | 16.5 | 92.1 |
| 40-44 | 159.3 | 18.7 | 93.3 |
| 45-49 | 165.6 | 19.3 | 92.1 |
| 50-54 | 161.1 | 18.7 | 89.6 |
| 55-59 | 142.3 | 16.4 | 85.1 |
| 60-64 | 107.0 | 11.6 | 71.7 |
| ≥65 | 0 | 0 | 41.9 |
| Female | | | |
| Age (years) | Average daily income  (USD) | Average hourly wage  (USD) | Employment rate (%) |
| 19 | 67.7 | 8.3 | 8.6 |
| 20-24 | 76.8 | 9.3 | 49.4 |
| 25-29 | 93.8 | 11.4 | 68.6 |
| 30-34 | 107.1 | 13.0 | 57.6 |
| 35-39 | 111.6 | 13.6 | 55.0 |
| 40-44 | 98.4 | 11.9 | 62.3 |
| 45-49 | 87.1 | 10.4 | 68.0 |
| 50-54 | 84.2 | 10.1 | 64.7 |
| 55-59 | 78.9 | 9.8 | 56.6 |
| 60-64 | 62.4 | 8.6 | 45.8 |
| ≥65 | 0 | 0 | 23.4 |

**Figure A. Incremental insurance-covered costs by age and the type of health care services.**


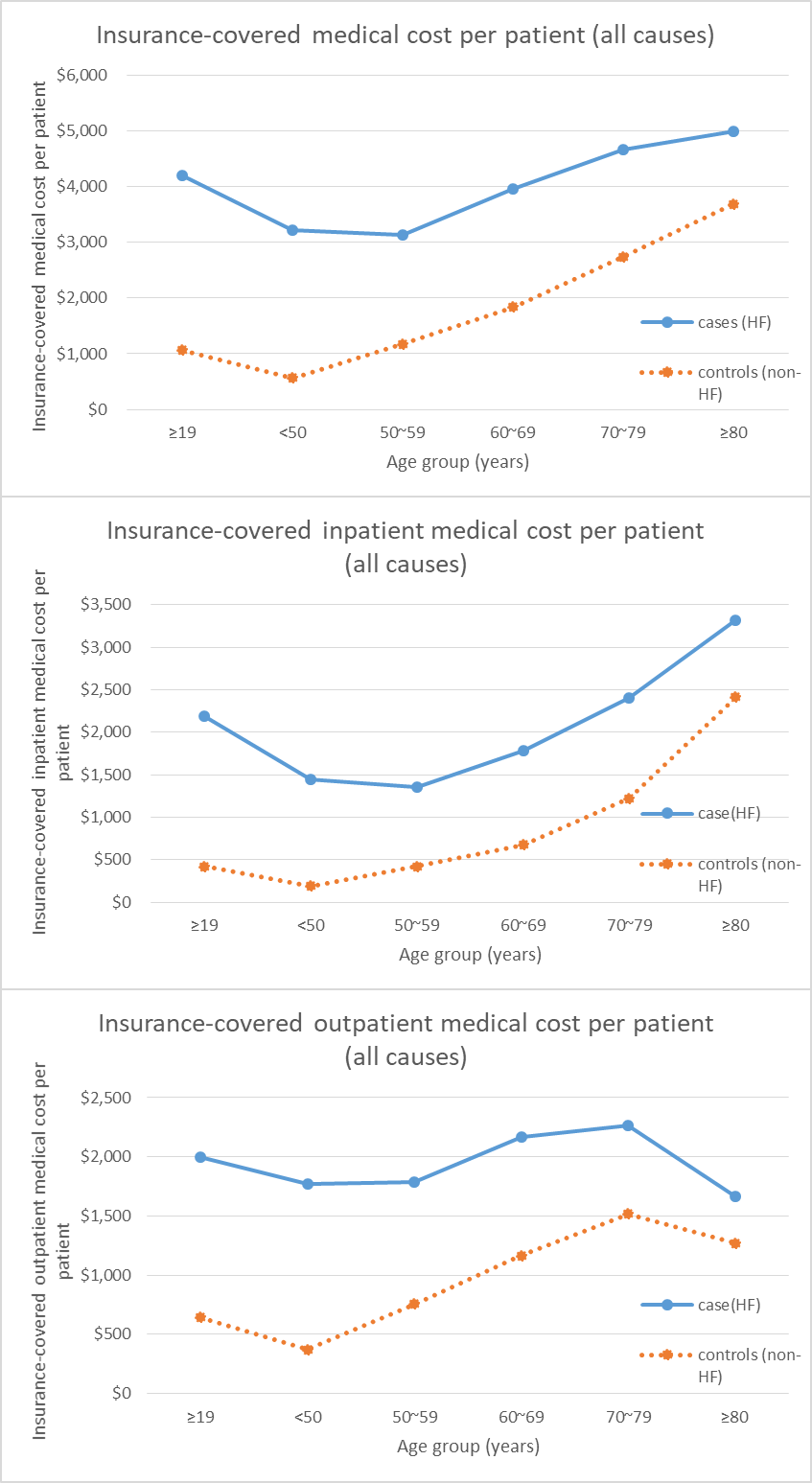


HF, heart failure.
